# Supplementary figures and images for: Susceptibility status of the wild-caught Phlebotomus argentipes (Diptera: Psychodidae: Phlebotominae), the sand fly vector of visceral leishmaniasis, to different insecticides in Nepal
Source: PLoS Negl Trop Dis. 2022 Jul 14;16(7):e0010304. doi: 10.1371/journal.pntd.0010304 (PMC9321455; doi:10.1371/journal.pntd.0010304)

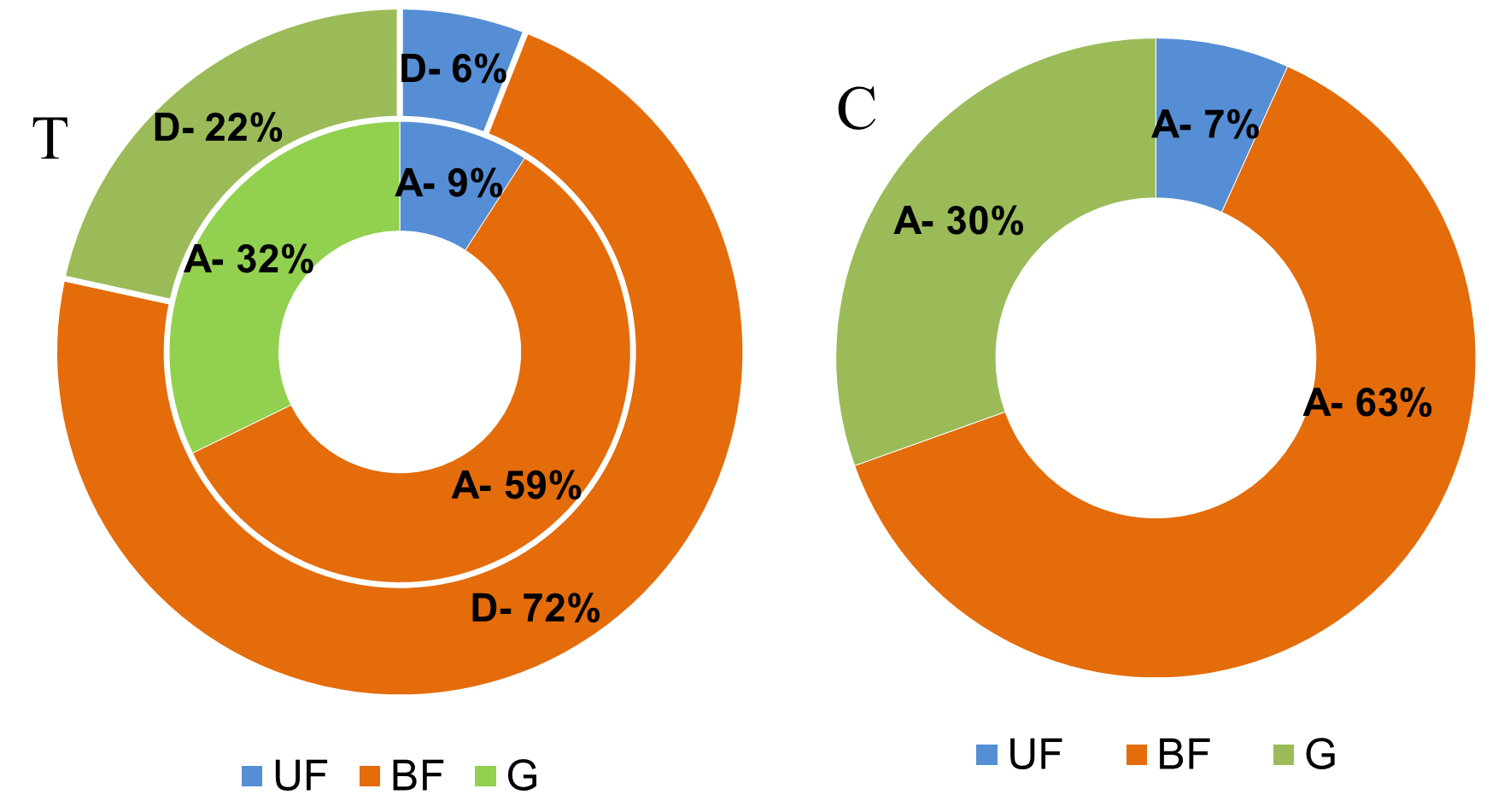

Supplement: S1 Fig — Inside the doughnut chart: A, alive and D, dead sand flies after 24h recovery period. UF, unfed; BF, blood-fed and G, gravid P. argentipes. (TIF) [file pntd.0010304.s001.tif]
