# Supplementary material for: Susceptibility status of the wild-caught Phlebotomus argentipes (Diptera: Psychodidae: Phlebotominae), the sand fly vector of visceral leishmaniasis, to different insecticides in Nepal
Source: PLoS Negl Trop Dis. 2022 Jul 14;16(7):e0010304. doi: 10.1371/journal.pntd.0010304 (PMC9321455; doi:10.1371/journal.pntd.0010304)
Supplement: S1 File — (DOC) [file pntd.0010304.s003.doc]

library(readxl)

dat <- read_excel("~/Lalita/DETAIL_DATA.xls")

dict <- read_excel("~/Lalita/DETAIL_DATA.xls", sheet="Variables")

dat$ID <- 1:nrow(dat)

insecticide_categorization <- setNames(

c("Pyrethroids",NA,NA,NA,"Carbamates","Organochlorines","Pyrethroids",NA,NA,NA,"Pyrethroids",NA,NA,NA,"Organophosphates",NA,NA,NA),

c("Alpha-cypermethrin","Alpha-cypermethrin alone","Alpha-cypermethrin_5X","Alpha-cypermethrin_R","Bendiocarb","DDT","Deltamethrin","Deltamethrin alone","Deltamethrin_5X","Deltamethrin_R","Lambda-cyhalothrin","Lambda-cyhalothrin alone","Lambda-cyhalothrin_5X","Lambda-cyhalothrin_R","Malathion","PBO+Alpha-cypermethrin","PBO+Deltamethrin","PBO+Lambda-cyhalothrin")

)

dat$Insecticides_cat <- factor(insecticide_categorization[dat$Insecticides], levels=c("Pyrethroids", "Carbamates", "Organophosphates"))

dat <- dat[dat$Phases == "Phase I",]

### Reshape data: type of fly from wide to long

dat_long <- dat[rep(1:nrow(dat), each=3),]

dat_long$DEAD_SUBTYPE <- NA

dat_long$ALIVE_SUBTYPE <- NA

dat_long$SUBTYPE <- factor(rep(c("PaU","PaF","PaG"), nrow(dat)), levels=c("PaU","PaF","PaG"))

dat_long$DEAD_SUBTYPE[dat_long$SUBTYPE == "PaU"] <- dat_long$`PaU_DEAD(IP)`[dat_long$SUBTYPE == "PaU"]

dat_long$DEAD_SUBTYPE[dat_long$SUBTYPE == "PaF"] <- dat_long$`PaF_DEAD(IP)`[dat_long$SUBTYPE == "PaF"]

dat_long$DEAD_SUBTYPE[dat_long$SUBTYPE == "PaG"] <- dat_long$`PaG_DEAD(IP)`[dat_long$SUBTYPE == "PaG"]

dat_long$ALIVE_SUBTYPE[dat_long$SUBTYPE == "PaU"] <- dat_long$`PaU_ALIVE(IP)`[dat_long$SUBTYPE == "PaU"]

dat_long$ALIVE_SUBTYPE[dat_long$SUBTYPE == "PaF"] <- dat_long$`PaF_ALIVE(IP)`[dat_long$SUBTYPE == "PaF"]

dat_long$ALIVE_SUBTYPE[dat_long$SUBTYPE == "PaG"] <- dat_long$`PaG_ALIVE(IP)`[dat_long$SUBTYPE == "PaG"]

dat_long$TOTAL_SUBTYPE <- dat_long$DEAD_SUBTYPE + dat_long$ALIVE_SUBTYPE

##### Models #####

# Simple GLMs

mod1 <- glm(DEAD ~ offset(log(SFLY_NUM)) + Village_type, family=poisson, data=dat)

mod2 <- glm(DEAD_SUBTYPE ~ offset(log(TOTAL_SUBTYPE)) + Village_type + SUBTYPE, family=poisson, subset=TOTAL_SUBTYPE > 0, data=dat_long)

# GLMs with mixed effects; results in boundary estimates

library(lme4)

mod3 <- glmer(DEAD_SUBTYPE ~ offset(log(TOTAL_SUBTYPE)) + (1|District/Village/REP_NUM) + Village_type + SUBTYPE, family=poisson, subset=TOTAL_SUBTYPE > 0, data=dat_long)

mod4 <- glmer(DEAD_SUBTYPE ~ offset(log(TOTAL_SUBTYPE)) + (1|Village) + Village_type + SUBTYPE, family=poisson, subset=TOTAL_SUBTYPE > 0, data=dat_long)

mod5 <- glmer(DEAD_SUBTYPE ~ offset(log(TOTAL_SUBTYPE)) + (1|District/Village/REP_NUM) + Village_type + SUBTYPE + Insecticides_cat, family=poisson, subset=TOTAL_SUBTYPE > 0, data=dat_long)

# Bayesian GLMs with default weakly informative priors

library(rstanarm)

#mods_1 <- stan_glmer(DEAD_SUBTYPE ~ offset(log(TOTAL_SUBTYPE)) + (1|District/Village/REP_NUM) + Village_type + SUBTYPE + Insecticides_cat, family=poisson, subset=TOTAL_SUBTYPE > 0, data=dat_long)

#mods_2 <- stan_glmer(DEAD_SUBTYPE ~ offset(log(TOTAL_SUBTYPE)) + (1|District/Village/REP_NUM) + Village_type + SUBTYPE + Insecticides, family=poisson, subset=TOTAL_SUBTYPE > 0, data=dat_long)

#mods_3 <- stan_glmer(DEAD_SUBTYPE ~ offset(log(TOTAL_SUBTYPE)) + (Village_type|District/Village/REP_NUM) + SUBTYPE + Insecticides, family=poisson, subset=TOTAL_SUBTYPE > 0, data=dat_long)

mods_4 <- stan_glmer(DEAD_SUBTYPE ~ offset(log(TOTAL_SUBTYPE)) + (1|District/Village/REP_NUM) + factor(Village_type, levels=c("NO-IRS","IRS")) * Insecticides + SUBTYPE, family=poisson, subset=TOTAL_SUBTYPE > 0, data=dat_long)

# mods_4a <- stan_glmer(DEAD_SUBTYPE ~ offset(log(TOTAL_SUBTYPE)) + (1|District/Village/REP_NUM) + factor(Village_type, levels=c("NO-IRS","IRS")) * Insecticides, family=poisson, subset=TOTAL_SUBTYPE > 0, data=dat_long)

#mods_4b <- stan_glmer(DEAD_SUBTYPE ~ offset(log(TOTAL_SUBTYPE)) + (1|District/Village/REP_NUM) + SUBTYPE, family=poisson, subset=TOTAL_SUBTYPE > 0, data=dat_long)

publish_fixef <- function(mod){

require(brms)

est <- fixef(mod)

int <- posterior_interval(mod)[1:length(est),]

test <- cbind(est, int)

test2 <- round(exp(test), digits=2)

pub <- paste0(test2[,1]," (",test2[,2]," - ",test2[,3],")")

return(cbind(pub))

}

# TABLE 2

####

publish_fixef(mods_4)

####

# Code for a now-unused table where the model was summarized with posterior predictions for every combination of the predictors; in each case, the posterior predictive distribution was summarized by taking the lambda (i.e. intercept) from a poisson regression model on the predictions

newdata <- data.frame(

Village_type=rep(c("NO-IRS","IRS"), each=5*3),

SUBTYPE=rep(rep(c("PaU","PaF","PaG"), each=5),2),

Insecticides=rep(unique(dat$Insecticides), 3*2),

TOTAL_SUBTYPE=1,

REP_NUM=1,

Village=1,

District=1

)

offsets <- log(newdata$TOTAL_SUBTYPE)

test <- posterior_predict(mods_4, newdata = newdata, re.form=NA, offset=offsets)

newdata$pred <- apply(test, 2, function(x) exp(coef(glm(x~ 1, family=poisson))))

#newdata$pred_test <- apply(test, 2, mean)

tapply(newdata$pred, list(newdata$Insecticides, newdata$SUBTYPE, newdata$Village_type), function(x)x)

##### Reshape data: survival #####

# Add count of censored cases

dat$KD_CENS <- dat$SFLY_NUM - dat$KD_60

kd_vars <- c("KD_00","KD_10","KD_15","KD_20","KD_30","KD_40","KD_50","KD_60")

kd_times <- c("00","10","15","20","30","40","50","60")

surv_temp1 <- dat[rep(1:nrow(dat), each=length(kd_vars)),]

surv_temp1$t <- rep(kd_times, nrow(dat))

surv_temp1$surv_KD <- NA

for (x in kd_times){

kd_var <- paste("KD",x, sep="_")

surv_temp1$surv_KD[surv_temp1$t == x] <- surv_temp1[[kd_var]][surv_temp1$t == x]

}

# KD counts are cumulative, so make un-cumulative

surv_temp1$surv_KD_diff <- ave(surv_temp1$surv_KD, surv_temp1$ID, FUN=function(x) c(x[[1]], diff(x)))

# Ultimately the KD weren't cumulative in the first place; there are 4 instances where fewer flies are downed in a later stage than in an earlier one. Pretend these stay downed

surv_temp1$surv_KD_diff[surv_temp1$surv_KD_diff < 0] <- 0

# expand so that there is one row per downed fly, plus censored cases

surv_temp1$censored_cases <- ifelse(surv_temp1$t == "60", surv_temp1$KD_CENS, 0)

surv_temp2 <- surv_temp1[rep(1:nrow(surv_temp1), surv_temp1$surv_KD_diff),]

surv_temp2$event <- 1

surv_temp3 <- surv_temp1[rep(1:nrow(surv_temp1), surv_temp1$censored_cases),]

surv_temp3$event <- 0

surv_final <- rbind(surv_temp2, surv_temp3)

surv_final_phaseI <- surv_final[surv_final$Phases == "Phase I",]

#### Recoding the insecticides name ###########################

surv_final_phaseI_I = surv_final_phaseI %>%

mutate (Insecticides = recode (Insecticides, "Alpha-cypermethrin" = "1. Alpha-cypermethrin 0.05%",

"Deltamethrin" = "2. Deltamethrin 0.05%", "Lambda-cyhalothrin" = "3. Lambda-cyhalothrin 0.05%",

"Bendiocarb" = "4. Bendiocarb 0.1%", "Malathion" = "5. Malathion 5%"))

##### Survival analysis #####

install.packages("survival")

library(survival)

##km <- survfit(Surv(as.numeric(t), event) ~ 1, data=surv_final)

##plot(km)

##cox_mod <- coxph(Surv(as.numeric(t), event) ~ Village_type + Insecticides, data=surv_final)

##cox_mod2 <- coxph(Surv(as.numeric(t), event) ~ Village_type * Insecticides, data=surv_final)

km2 <- survfit(Surv(as.numeric(t), event) ~ Village_type + Insecticides, data=surv_final_phaseI_I)

plot(km2)

##################### installing package "survminer" #########################

install.packages("survminer")

library(survminer)

ggsurvplot(km, conf.int=TRUE, color="black")

ggsurvplot(km2, conf.int=FALSE, linetype=c(rep("solid",5),rep("dashed",5))) + theme_grey()

#FIG. 4

####

survplot <- ggsurvplot_facet(km2, data=surv_final_phaseI_I,facet.by = "Insecticides",

color ="black", linetype="Village_type",

xlab = "Time in minutes", ylab = "Proportion not knocked down", font.x = c(14, "bold"), font.y = c(14, "bold"),

short.panel.labs = TRUE, panel.labs.font= list(size=12, color="black"))

survplot

#### saving the figure in higher resolution (publication grade)###################

ggsave("survplot.tiff", dpi = 300)

ggsave("survplot_jpg.jpg", dpi = 300)

####

ggsurvplot_facet(km2, data=surv_final, facet.by = "Village_type", color="black", linetype="Insecticides")

############################################################################################

###################### Knockdown time calculation (KDT50 and KDT99) using package "ecotox" ##################

#### install packages "tidyverse" and "ecotox"

##install.packages("ecotox")

install.packages("ecotox", repos="https://CRAN.R-project.org")

library(ecotox)

##install.packages("tidyverse")

install.packages("tidyverse")

library(tidyverse)

##setting working directory

setwd("E:/2. RESISTANCE TEST_2019_20/DATA/ANALYSIS/ANALYSIS_R/kdt_analysis/KDT50_95")

getwd()

##list files

list.files()

##Read the CSV data

df <- read.csv("DETAIL_DATA.csv")

view(df)

head(df)

str(df$CORR_MORT_percent)

hist(df$CORR_MORT_percent)

### Subset with select argument, a new dataframe with desired columns only

df1 = df %>% select (Village, Phases, Village_type, Insecticides, CONC, SFLY_NUM, KD_10_,KD_15,

KD_20, KD_30, KD_40, KD_50, KD_60)

## gathering columns (KD_10_,KD_15,KD_20, KD_30, KD_40, KD_50, KD_60) to make long table

## with new columns 'KDT' for knock down time and 'Response' for the number of sand flies knocked down

df1_long= gather(df1,KDT,Response,7:13)

head(df1_long)

## Recoding the categories into numbers under the column KDT in dataframe 'df1_long'

df1_long_recode = df1_long %>% mutate (KDT = recode (KDT, KD_10_ = "10", KD_15 = "15",

KD_20 = "20", KD_30 = "30", KD_40 = "40",

KD_50 = "50", KD_60 = "60"))

### converting KDT variable structure from 'character' to 'integer'

df1_long_recode$KDT = as.integer(df1_long_recode$KDT)

## checking the structure of the variables in the new dataframe

str(df1_long_recode)

### Exporting new dataframe into xlsx file

write_xlsx(df1_long_recode, "E:/2. RESISTANCE TEST_2019_20/DATA/ANALYSIS/ANALYSIS_R/kdt_analysis/KDT50_95//DATA_long_KDT.xlsx")

#################################################### calculation of kdt50 and kdt 99 by probit analysis ###########################

### 1. Calculation of kdt50 and kdt99 for 'Phase I', 'Alpha-cypermethrin' in IRS villages from dataframe 'df1_long_recode'

IRS1_Alpha_kdt5099= df1_long_recode %>% filter (Phases == "Phase I", Village_type == "IRS",

Insecticides == "Alpha-cypermethrin") %>%

ecotox::LT_probit(formula = (Response/SFLY_NUM) ~ log10(KDT),

p = c(50,99),

weights = SFLY_NUM)

### Exporting new dataframe into xlsx file

write_xlsx(IRS1_Alpha_kdt5099, "E:/2. RESISTANCE TEST_2019_20/DATA/ANALYSIS/ANALYSIS_R/kdt_analysis/KDT50_95/Results//IRS1_Alpha_kdt5099.xlsx")

#################################################################################################################

### 2. Calculation of kdt50 and kdt99 for 'Phase I', 'Deltamethrin' in IRS villages from dataframe 'df1_long_recode'

IRS1_Delta_kdt5099= df1_long_recode %>% filter (Phases == "Phase I", Village_type == "IRS", Insecticides == "Deltamethrin") %>%

ecotox::LT_probit(formula = (Response/SFLY_NUM) ~ log10(KDT),

p = c(50,99),

weights = SFLY_NUM)

### Exporting new dataframe into xlsx file

write_xlsx(IRS1_Delta_kdt5099, "E:/2. RESISTANCE TEST_2019_20/DATA/ANALYSIS/ANALYSIS_R/kdt_analysis/KDT50_95/Results//IRS1_Delta_kdt5099.xlsx")

#######################################################################################################################

### 3. Calculation of kdt50 and kdt99 for 'Phase I', 'Lambda-cyhalothrin' in IRS villages from dataframe 'df1_long_recode'

IRS1_Lambda_kdt5099= df1_long_recode %>% filter (Phases == "Phase I", Village_type == "IRS", Insecticides == "Lambda-cyhalothrin") %>%

ecotox::LT_probit(formula = (Response/SFLY_NUM) ~ log10(KDT),

p = c(50,99),

weights = SFLY_NUM)

### Exporting new dataframe into xlsx file

write_xlsx(IRS1_Lambda_kdt5099, "E:/2. RESISTANCE TEST_2019_20/DATA/ANALYSIS/ANALYSIS_R/kdt_analysis/KDT50_95/Results//IRS1_Lambda_kdt5099.xlsx")

##################################################################################################################

### 4. Calculation of kdt50 and kdt99 for 'Phase I', 'Bendiocarb' in IRS villages from dataframe 'df1_long_recode'

IRS1_Bendiocarb_kdt5099 = df1_long_recode %>% filter (Phases == "Phase I", Village_type == "IRS", Insecticides == "Bendiocarb") %>%

ecotox::LT_probit(formula = (Response/SFLY_NUM) ~ log10(KDT),

p = c(50,99),

weights = SFLY_NUM)

### Exporting new dataframe into xlsx file

write_xlsx(IRS1_Bendiocarb_kdt5099, "E:/2. RESISTANCE TEST_2019_20/DATA/ANALYSIS/ANALYSIS_R/kdt_analysis/KDT50_95/Results//IRS1_Bendiocarb_kdt5099.xlsx")

##############################################################################################################################

### 5. Calculation of kdt50 and kdt99 for 'Phase I', 'Malathion' in IRS villages from dataframe 'df1_long_recode'

IRS1_Malathion_kdt5099 = df1_long_recode %>% filter (Phases == "Phase I", Village_type == "IRS", Insecticides == "Malathion") %>%

ecotox::LT_probit(formula = (Response/SFLY_NUM) ~ log10(KDT),

p = c(50,99),

weights = SFLY_NUM)

### Exporting new dataframe into xlsx file

write_xlsx(IRS1_Malathion_kdt5099, "E:/2. RESISTANCE TEST_2019_20/DATA/ANALYSIS/ANALYSIS_R/kdt_analysis/KDT50_95/Results//IRS1_Malathion_kdt5099.xlsx")

#################################################################################################################

### 6. Calculation of kdt50 and kdt99 for 'Phase I', 'Alpha-cypermethrin' in NO-IRS villages from dataframe 'df1_long_recode'

NOIRS1_Alpha_kdt5099= df1_long_recode %>% filter (Phases == "Phase I", Village_type == "NO-IRS", Insecticides == "Alpha-cypermethrin") %>%

ecotox::LT_probit(formula = (Response/SFLY_NUM) ~ log10(KDT),

p = c(50,99),

weights = SFLY_NUM)

### Exporting new dataframe into xlsx file

write_xlsx(NOIRS1_Alpha_kdt5099, "E:/2. RESISTANCE TEST_2019_20/DATA/ANALYSIS/ANALYSIS_R/kdt_analysis/KDT50_95/Results//NOIRS1_Alpha_kdt5099.xlsx")

#################################################################################################################

### 7. Calculation of kdt50 and kdt99 for 'Phase I', 'Deltamethrin' in NO-IRS villages from dataframe 'df1_long_recode'

NOIRS1_Delta_kdt5099= df1_long_recode %>% filter (Phases == "Phase I", Village_type == "NO-IRS", Insecticides == "Deltamethrin") %>%

ecotox::LT_probit(formula = (Response/SFLY_NUM) ~ log10(KDT),

p = c(50,99),

weights = SFLY_NUM)

### Exporting new dataframe into xlsx file

write_xlsx(NOIRS1_Delta_kdt5099, "E:/2. RESISTANCE TEST_2019_20/DATA/ANALYSIS/ANALYSIS_R/kdt_analysis/KDT50_95/Results//NOIRS1_Delta_kdt5099.xlsx")

#######################################################################################################################

### 8. Calculation of kdt50 and kdt99 for 'Phase I', 'Lambda-cyhalothrin' in NO-IRS villages from dataframe 'df1_long_recode'

NOIRS1_Lambda_kdt5099= df1_long_recode %>% filter (Phases == "Phase I", Village_type == "NO-IRS", Insecticides == "Lambda-cyhalothrin") %>%

ecotox::LT_probit(formula = (Response/SFLY_NUM) ~ log10(KDT),

p = c(50,99),

weights = SFLY_NUM)

### Exporting new dataframe into xlsx file

write_xlsx(NOIRS1_Lambda_kdt5099, "E:/2. RESISTANCE TEST_2019_20/DATA/ANALYSIS/ANALYSIS_R/kdt_analysis/KDT50_95/Results//NOIRS1_Lambda_kdt5099.xlsx")

##################################################################################################################

### 9. Calculation of kdt50 and kdt99 for 'Phase I', 'Bendiocarb' in NO-IRS villages from dataframe 'df1_long_recode'

NOIRS1_Bendiocarb_kdt5099 = df1_long_recode %>% filter (Phases == "Phase I", Village_type == "NO-IRS", Insecticides == "Bendiocarb") %>%

ecotox::LT_probit(formula = (Response/SFLY_NUM) ~ log10(KDT),

p = c(50,99),

weights = SFLY_NUM)

### Exporting new dataframe into xlsx file

write_xlsx(NOIRS1_Bendiocarb_kdt5099, "E:/2. RESISTANCE TEST_2019_20/DATA/ANALYSIS/ANALYSIS_R/kdt_analysis/KDT50_95/Results//NOIRS1_Bendiocarb_kdt5099.xlsx")

##############################################################################################################################

### 10. Calculation of kdt50 and kdt99 for 'Phase I', 'Malathion' in NO-IRS villages from dataframe 'df1_long_recode'

NOIRS1_Malathion_kdt5099 = df1_long_recode %>% filter (Phases == "Phase I", Village_type == "NO-IRS", Insecticides == "Malathion") %>%

ecotox::LT_probit(formula = (Response/SFLY_NUM) ~ log10(KDT),

p = c(50,99),

weights = SFLY_NUM)

### Exporting new dataframe into xlsx file

write_xlsx(NOIRS1_Malathion_kdt5099, "E:/2. RESISTANCE TEST_2019_20/DATA/ANALYSIS/ANALYSIS_R/kdt_analysis/KDT50_95/Results//NOIRS1_Malathion_kdt5099.xlsx")

#######################################################################################################################

### 11. Calculation of kdt50 and kdt99 for 'DDT' in IRS villages from dataframe 'df1_long_recode'

IRS2_DDT_kdt5099 = df1_long_recode %>% filter (Phases == "Phase II", Village_type == "IRS", Insecticides == "DDT") %>%

ecotox::LT_probit(formula = (Response/SFLY_NUM) ~ log10(KDT),

p = c(50,99),

weights = SFLY_NUM)

### Exporting new dataframe into xlsx file

write_xlsx(IRS2_DDT_kdt5099, "E:/2. RESISTANCE TEST_2019_20/DATA/ANALYSIS/ANALYSIS_R/kdt_analysis/KDT50_95/Results//IRS2_DDT_kdt5099.xlsx")

#########################################################################################################################

### 12. Calculation of kdt50 and kdt99 for 'DDT' in NO-IRS villages from dataframe 'df1_long_recode'

NOIRS2_DDT_kdt5099 = df1_long_recode %>% filter (Phases == "Phase II", Village_type == "NO-IRS", Insecticides == "DDT") %>%

ecotox::LT_probit(formula = (Response/SFLY_NUM) ~ log10(KDT),

p = c(50,99),

weights = SFLY_NUM)

### Exporting new dataframe into xlsx file

write_xlsx(NOIRS2_DDT_kdt5099, "E:/2. RESISTANCE TEST_2019_20/DATA/ANALYSIS/ANALYSIS_R/kdt_analysis/KDT50_95/Results//NOIRS2_DDT_kdt5099.xlsx")

#######################################################################################################################

### 13. Calculation of kdt50 and kdt99 for 'Alpha-cypermethrin_5X' in IRS villages from dataframe 'df1_long_recode'

IRS2_Alpha5X_kdt5099 = df1_long_recode %>% filter (Phases == "Phase II", Village_type == "IRS", Insecticides == "Alpha-cypermethrin_5X") %>%

ecotox::LT_probit(formula = (Response/SFLY_NUM) ~ log10(KDT),

p = c(50,99),

weights = SFLY_NUM)

### Exporting new dataframe into xlsx file

write_xlsx(IRS2_Alpha5X_kdt5099, "E:/2. RESISTANCE TEST_2019_20/DATA/ANALYSIS/ANALYSIS_R/kdt_analysis/KDT50_95/Results//IRS2_Alpha5X_kdt5099.xlsx")

############################################################################################################################

### 14. Calculation of kdt50 and kdt99 for 'Deltamethrin_5X' in IRS villages from dataframe 'df1_long_recode'

IRS2_Delta5X_kdt5099 = df1_long_recode %>% filter (Phases == "Phase II", Village_type == "IRS", Insecticides == "Deltamethrin_5X") %>%

ecotox::LT_probit(formula = (Response/SFLY_NUM) ~ log10(KDT),

p = c(50,99),

weights = SFLY_NUM)

### Exporting new dataframe into xlsx file

write_xlsx(IRS2_Delta5X_kdt5099, "E:/2. RESISTANCE TEST_2019_20/DATA/ANALYSIS/ANALYSIS_R/kdt_analysis/KDT50_95/Results//IRS2_Delta5X_kdt5099.xlsx")

############################################################################################################################

### 15. Calculation of kdt50 and kdt99 for 'Lambda-cyhalothrin_5X' in IRS villages from dataframe 'df1_long_recode'

IRS2_Lambda5X_kdt5099 = df1_long_recode %>% filter (Phases == "Phase II", Village_type == "IRS", Insecticides == "Lambda-cyhalothrin_5X") %>%

ecotox::LT_probit(formula = (Response/SFLY_NUM) ~ log10(KDT),

p = c(50,99),

weights = SFLY_NUM)

### Exporting new dataframe into xlsx file

write_xlsx(IRS2_Lambda5X_kdt5099, "E:/2. RESISTANCE TEST_2019_20/DATA/ANALYSIS/ANALYSIS_R/kdt_analysis/KDT50_95/Results//IRS2_Lambda5X_kdt5099.xlsx")

######################################################################################################################################33

################## Barplot for the mortality assessment ################################

####### Fig 3 ################

library(tidyverse)

df <- read.csv("DETAIL_DATA.csv")

View(df)

head(df)

### subsetting the data for phase I

df_phaseI = filter(df, Phases == "Phase I")

###Calculation of mean, standard deviation, standard error and upper and lower limits of Corrected mortality percentage

df_phaseI_sumzd = group_by(df_phaseI, Insecticides, Village_type, Village) %>%

dplyr::summarise(mean = mean(CORR_MORT_percent), sd = sd(CORR_MORT_percent), N_rep=n(),

se = sd/sqrt(N_rep), upper_limit = mean+se, lower_limit = mean-se)

####installing package (writexl)

install.packages("writexl")

library(writexl)

###saving the summary in excel file

write_xlsx(df_phaseI_sumzd, "E:/2. RESISTANCE TEST_2019_20/DATA/ANALYSIS/ANALYSIS_R/Trial_graph//df_phaseI_sumzd.xlsx")

### Renaming the categories of column 'Insecticides'

df_phaseI_sumzd_rename = df_phaseI_sumzd %>%

mutate (Insecticides = recode(Insecticides, "Alpha-cypermethrin" = "Alpha-cypermethrin 0.05%",

"Deltamethrin" = "Deltamethrin 0.05%",

"Lambda-cyhalothrin" = "Lambda-cyhalothrin 0.05%", "Bendiocarb" = "Bendiocarb 0.1%", "Malathion" = "Malathion 5%"))

### Renaming the categories of column 'Village_type'

df_phaseI_sumzd_rename = df_phaseI_sumzd_rename %>% mutate (Village_type = recode (Village_type, "NO-IRS" = "Non-IRS villages", "IRS" = "IRS villages"))

df_phaseI_sumzd_rename

##### Rearranging the sequence of Insecticides

### Insecticide category from alphabetical order to desired order

df_phaseI_sumzd_rename_rearzd = df_phaseI_sumzd_rename %>%

group_by(Village_type) %>%

mutate(Insecticides = fct_relevel(Insecticides,

c("Alpha-cypermethrin 0.05%",

"Deltamethrin 0.05%",

"Lambda-cyhalothrin 0.05%",

"Bendiocarb 0.1%",

"Malathion 5%")))

View(df_phaseI_sumzd_rename_rearzd)

## Rearranging the sequence of village in IRS and Non-IRS villages

df_phaseI_sumzd_rename_rearzd1 = df_phaseI_sumzd_rename_rearzd %>% group_by(Village_type) %>%

mutate(Village = fct_relevel(Village,

c("Majhare","Belhichapena",

"Bishanpur","Madhepura",

"Riterkhor", "Pokhariya",

"G.Parwaha","Dahipaudi",

"Anarban","Lodba")))

df1=df_phaseI_sumzd_rename_rearzd1

df1

##saving the updated summary table in excel file

write_xlsx(df1, "E:/2. RESISTANCE TEST_2019_20/DATA/ANALYSIS/ANALYSIS_R/Trial_graph//phaseI_summary.xlsx")

## spliting the facet"

df1s = split(df1, f = df1$Village_type)

### barplot of desired dataframe for IRS villages

bp1= ggplot(df1s$"IRS villages", aes(x =Insecticides,y=mean, fill= Village, label=mean))+

geom_bar(stat="identity", color="black", position = position_dodge(0.9), width = 0.8)+

## Reduce saturation (chromaticity) from 100 to 50 and increase luminance

scale_fill_hue(c=70, l=70)+

## Adding reference line in the barplot

geom_hline(yintercept = 90, linetype = "dashed", color="red", size = 0.75)+

geom_hline(yintercept = 98, linetype = "dashed", color="darkgreen", size = 0.75)+

##Creating the barplot without space between X-axis labels and the bars & setting y axis range

scale_y_continuous(breaks=c(0,10,20,30,40,50,60,70,80,90,100,110),

expand = c(0,0))+

## Adding error bars with standard error value

geom_errorbar(aes(ymin=mean-se, ymax=mean+se),

width = 0.2, size = 0.703,

position = position_dodge(0.9), alpha=0.7)+

## Changing theme of the barplot area

theme_classic ()+

## editing appearance of facet level

## theme(strip.text = element_text(size = 14),

## strip.background = element_rect(color = NA, size = 3),

## strip.placement = "outside")+

##editing appearance of theme such as title, axis labels, legend

theme(plot.title = element_text (size = 16, hjust = 0.5, vjust = 2),

axis.text=element_text(size=11, color = "black"),

axis.title=element_text(size=14),

legend.title = element_blank(),

legend.text=element_text(size = 12),

legend.position = "bottom", legend.direction = "horizontal",

legend.key.size = unit(0.8, "lines"),

legend.key.width = unit(0.3, "cm"),

legend.key.height = unit(0.3, "cm"),

legend.box.margin = margin(0,0,0,0, "cm"),

legend.box.spacing = unit (-0.5, "cm"))+

##change plot titles using the function labs()

labs(x="", y = "Mortality percentage", title = "IRS villages")

### Final barplot for mean mortality after 24 hours of bioassay

bp1

###############################################################

####For Non-IRS villages##########################

bp3 = ggplot(df1s$"Non-IRS", aes(x =Insecticides,y=mean, fill= Village, label=mean))+

geom_bar(stat="identity", color="black", position = position_dodge(0.9), width = 0.8)+

## bars with desired color

scale_fill_manual(values = c("Pokhariya" = "lightpink2",

"G.Parwaha" = "lightgoldenrod3",

"Dahipaudi" = "cyan1",

"Anarban" = "cyan4",

"Lodba" = "dodgerblue3"))+

## Reduce saturation (chromaticity) from 100 to 50 and increase luminance

## scale_fill_hue(c=70, l=70)+

## Adding reference line in the barplot

geom_hline(yintercept = 90, linetype = "dashed", color="red", size = 0.75)+

geom_hline(yintercept = 98, linetype = "dashed", color="darkgreen", size = 0.75)+

##Creating the barplot without space between X-axis labels and the bars & setting y axis range

scale_y_continuous(breaks=c(0,10,20,30,40,50,60,70,80,90,100,110),

expand = c(0,0))+

## Adding error bars with standard error value

geom_errorbar(aes(ymin=mean-se, ymax=mean+se),

width = 0.2, size = 0.703,

position = position_dodge(0.9), alpha=0.7)+

## Creating facets of Village type

##facet_wrap (~ Village_type, ncol = 2)+

## Changing theme of the barplot area

theme_classic ()+

## editing appearance of facet level

## theme(strip.text = element_text(size = 14),

## strip.background = element_rect(color = NA, size = 3),

## strip.placement = "outside")+

##editing appearance of theme such as title, axis labels, legend

theme(plot.title = element_text (size = 16, hjust = 0.5, vjust = 2),

axis.text=element_text(size=11, color = "black"),

axis.title=element_text(size=14),

legend.title = element_blank(),

legend.text=element_text(size = 12),

legend.position = "bottom", legend.direction = "horizontal",

legend.key.size = unit(0.8, "lines"),

legend.key.width = unit(0.3, "cm"),

legend.key.height = unit(0.3, "cm"),

legend.box.margin = margin(0,0,0,0, "cm"),

legend.box.spacing = unit (-0.5, "cm"))+

##change plot titles using the function labs()

labs(x="", y = "Mortality percentage", title = "Non-IRS villages")

### Final barplot for mean mortality after 24 hours of bioassay

bp3

library(ggpubr)

ggarrange(bp1, bp3,labels = c("A","B"), ncol=1, nrow =2)

ggsave("Mortality_IRSnonIRS.tiff", dpi = 500) #### Fig 3

ggsave("Mortality_IRSnonIRS_jpg.jpg", dpi = 500)

###############################################################################################3

##### Fig 5 ###################

##### Bar plot for Synergist test for selected insecticides in selected villages #####

## Selecting the desired rows

df_synergist = df%>% filter(Village %in% c("Belhichapena","Bishanpur","Riterkhor")) %>%

filter(Insecticides %in% c("Alphacypermethrin alone",

"Deltamethrin alone", "Lambda-cyhalothrin alone",

"PBO+Alpha-cypermethrin", "PBO+Deltamethrin",

"PBO+Lambda-cyhalothrin"))

### Rename 'Alphacypermethrin alone' to 'Alpha-cypermethrin alone'

df_synergist = df_synergist %>% mutate(Insecticides = recode(Insecticides, "Alpha-cypermethrin alone" = "Alpha-cypermethrin 0.05% alone",

"Deltamethrin alone" = "Deltamethrin 0.05% alone", "Lambda-cyhalothrin alone" = "Lambda-cyhalothrin 0.05% alone",

"PBO+Alpha-cypermethrin"= "PBO 4% + Alpha-cypermethrin 0.05%",

"PBO+Deltamethrin" = "PBO 4% + Deltamethrin 0.05%","PBO+Lambda-cyhalothrin" = "PBO 4% + Lambda-cyhalothrin 0.05%"))

View(df_synergist)

###Calculation of mean, standard deviation, standard error and upper and lower limits of mortality percentage

df_synergist_sumzd = group_by(df_synergist, Insecticides, Village) %>%

dplyr::summarise(mean = mean(CORR_MORT_percent), sd = sd(CORR_MORT_percent), N_rep=n(),

se = sd/sqrt(N_rep), upper_limit = mean+se, lower_limit = mean-se)

###saving the data for synergist test file in excel file

df_synergist_sumzd = write_xlsx(df_synergist_sumzd, "E:/2. RESISTANCE TEST_2019_20/DATA/ANALYSIS/ANALYSIS_R/Bargraphs/barplot_analysis//Synergist_pyrethroid_sumzd.xlsx")

### Adding '0' values for "Deltamethrin" and "Lambda-cyhalothrin" in Belhichapena and Riterkhor

##New dataframe is

##Read the CSV data

df_synergist_sumzd1 <- read.csv("Synergist_pyrethroid_sumzd_add.csv")

### Releveling the sequence of Insecticides in table and in barplot

df_synergist_sumzd1 = df_synergist_sumzd1 %>% mutate(Insecticides = fct_relevel(Insecticides, c("Alpha-cypermethrin 0.05% alone",

"PBO 4% + Alpha-cypermethrin 0.05%",

"Deltamethrin 0.05% alone",

"PBO 4% + Deltamethrin 0.05%",

"Lambda-cyhalothrin 0.05% alone",

"PBO 4% + Lambda-cyhalothrin 0.05%")))

## barplot of desired dataframe

bp_df_synergist = ggplot(df_synergist_sumzd1, aes(x = Village, y=mean, fill = Insecticides, labels = mean))+

geom_bar(stat="identity", color="black", position = position_dodge (0.7), width = 0.6)+

## bars with desired color

scale_fill_manual(values = c("Alpha-cypermethrin 0.05% alone" = "deepskyblue",

"PBO 4% + Alpha-cypermethrin 0.05%" = "deepskyblue4",

"Deltamethrin 0.05% alone" = "cadetblue1",

"PBO 4% + Deltamethrin 0.05%" = "cadetblue4",

"Lambda-cyhalothrin 0.05% alone" = "darkseagreen2",

"PBO 4% + Lambda-cyhalothrin 0.05%" = "darkseagreen4"))+

## Adding reference line in the barplot

geom_hline(yintercept = 90, linetype = "dashed", color="red", size = 0.75)+

geom_hline(yintercept = 98, linetype = "dashed", color="darkgreen", size = 0.75)+

##Creating the barplot without space between X-axis labels and the bars & setting y axis range

scale_y_continuous(limits=c(0,100), breaks=c(0,10,20,30,40,50,60,70,80,90,100),

expand = c(0,0))+

## Adding error bars with standard error value

geom_errorbar(aes(ymin=mean-se, ymax=mean+se), width = 0.2, size = 0.8,

position = position_dodge(0.7), alpha=0.7)+

theme_classic ()+

##editing appearance of theme such as title, axis labels, legend

theme(axis.text=element_text(size=11, color = "black"),

axis.title=element_text(size=14),

legend.title = element_blank(),

legend.text=element_text(size = 12), ###, face ="bold")

legend.position = "bottom", legend.direction = "horizontal",

legend.key.size = unit(0.8, "lines"),

legend.key.width = unit(0.3, "cm"),

legend.key.height = unit(0.3, "cm"),

legend.box.margin = margin(0,0,0,0, "cm"),

legend.box.spacing = unit (-0.5, "cm"))+

##change plot titles using the function labs()

labs(x="", y = "Mortality percentage")

## Final barplot for synergist test

bp_df_synergist

ggsave("synergist_pyrethroid.tiff", dpi = 350)

ggsave("synergist_pyrethroid_jpg.jpg", dpi = 350)

###################################################################################

###### Fig 6 ##############

### Bar plot for DDT and pyerethroid test for selected insecticides in selected villages

## Selecting the desired rows

df_DDT_Pyrethroid = df%>% filter(Village %in% c("Bishanpur","Riterkhor", "Lodba")) %>%

filter(Insecticides %in% c("Alpha-cypermethrin",

"Deltamethrin", "Lambda-cyhalothrin",

"DDT"))

###Calculation of mean, standard deviation, standard error and upper and lower limits of mortality percentage

df_DDT_Pyrethroid_sumzd = group_by(df_DDT_Pyrethroid, Insecticides, Village_type, Village) %>%

dplyr::summarise(mean = mean(CORR_MORT_percent), sd = sd(CORR_MORT_percent), N_rep=n(),

se = sd/sqrt(N_rep), upper_limit = mean+se, lower_limit = mean-se)

### Rename 'Alpha-cypermethrin' to 'Alpha-cypermethrin 0.05%'

df_DDT_Pyrethroid_sumzd = df_DDT_Pyrethroid_sumzd %>% mutate(Insecticides = recode(Insecticides, "Alpha-cypermethrin" = "Alpha-cypermethrin 0.05%",

"Deltamethrin" = "Deltamethrin 0.05%", "Lambda-cyhalothrin" = "Lambda-cyhalothrin 0.05%",

"DDT" = "DDT 4%"))

df_DDT_Pyrethroid_sumzd

### Releveling the sequence of Insecticides in table and in barplot

df_DDT_Pyrethroid_sumzd = df_DDT_Pyrethroid_sumzd %>% mutate(Village = fct_relevel(Village, c("Bishanpur", "Riterkhor", "Lodba")))

df_DDT_Pyrethroid_sumzd1 = df_DDT_Pyrethroid_sumzd %>% group_by (Village_type)%>% mutate (Insecticides = fct_relevel(Insecticides, c("DDT 4%", "Alpha-cypermethrin 0.05%", "Deltamethrin 0.05%",

"Lambda-cyhalothrin 0.05%")))

## barplot of desired dataframe

bp_DDT_Pyrethroid = ggplot(df_DDT_Pyrethroid_sumzd1, aes(x = Village, y=mean, fill = Insecticides, labels = mean))+

geom_bar(stat="identity", color="black", position = position_dodge (0.8), width = 0.7)+

## Reduce saturation (chromaticity) from 100 to 50 and increase luminance

scale_fill_hue(c=70, l=70)+

## Adding reference line in the barplot

geom_hline(yintercept = 90, linetype = "dashed", color="red", size = 0.75)+

geom_hline(yintercept = 98, linetype = "dashed", color="darkgreen", size = 0.75)+

##Creating the barplot without space between X-axis labels and the bars & setting y axis range

scale_y_continuous(limits=c(0,100), breaks=c(0,10,20,30,40,50,60,70,80,90,100),

expand = c(0,0))+

## Adding error bars with standard error value

geom_errorbar(aes(ymin=mean-se, ymax=mean+se), width = 0.2, size = 0.8,

position = position_dodge(0.8), alpha=0.7)+

theme_classic ()+

##editing appearance of theme such as title, axis labels, legend

theme(axis.text=element_text(size=11, color = "black"),

axis.title=element_text(size=14),

legend.title = element_blank(),

legend.text=element_text(size = 12), ##, face ="bold"),

legend.position = "bottom", legend.direction = "horizontal",

legend.key.size = unit(0.8, "lines"),

legend.key.width = unit(0.3, "cm"),

legend.key.height = unit(0.3, "cm"),

legend.box.margin = margin(0,0,0,0, "cm"),

legend.box.spacing = unit (-0.5, "cm"))+

##change plot titles using the function labs()

labs(x="", y = "Mortality percentage")

bp_DDT_Pyrethroid

ggsave("DDT_pyrethroid.tiff", dpi = 400)

ggsave("DDT_pyrethroid_jpg.jpg", dpi = 400)
